# Preliminary Observations of Population Genetics and Relatedness of the Broadnose Sevengill Shark, *Notorynchus cepedianus*, in Two Northeast Pacific Estuaries

**DOI:** 10.1371/journal.pone.0129278

**Published:** 2015-06-08

**Authors:** Shawn Larson, Debbie Farrer, Dayv Lowry, David A. Ebert

**Affiliations:** 1 Seattle Aquarium, Seattle, Washington, United States of America; 2 Washington Department of Fish and Wildlife, Washington, United States of America; 3 Pacific Shark Research Center, Moss Landing Marine Laboratories, Moss Landing, California, United States of America; Macquarie University, AUSTRALIA

## Abstract

The broadnose sevengill shark, *Notorynchus cepedianus*, a common coastal species in the eastern North Pacific, was sampled during routine capture and tagging operations conducted from 2005–2012. One hundred and thirty three biopsy samples were taken during these research operations in Willapa Bay, Washington and in San Francisco Bay, California. Genotypic data from seven polymorphic microsatellites (derived from the related sixgill shark, *Hexanchus griseus*) were used to describe *N*. *cepedianus* genetic diversity, population structure and relatedness. Diversity within *N*. *cepedianus* was found to be low to moderate with an average observed heterozygosity of 0.41, expected heterozygosity of 0.53, and an average of 5.1 alleles per microsatellite locus. There was no evidence of a recent population bottleneck based on genetic data. Analyses of genetic differences between the two sampled estuaries suggest two distinct populations with some genetic mixing of sharks sampled during 2005–2006. Relatedness within sampled populations was high, with percent relatedness among sharks caught in the same area indicating 42.30% first-order relative relationships (full or half siblings). Estuary-specific familial relationships suggest that management of *N*. *cepedianus* on the U.S. West Coast should incorporate stock-specific management goals to conserve this ecologically important predator.

## Introduction

Sharks are predators found in every ocean of the world. They tend to mature slowly and have low reproductive rates, making many species extremely vulnerable to exploitation [1–5]. Of the over 1,000 chondrichthyan fishes—sharks, rays, and chimaeras, it is estimated that 1 in 4 are threatened according to IUCN Red List criteria due to overfishing and/or incidental take [6, 7]. Effective management of most shark species has proven difficult not only because of their life history traits but also because much remains unknown about their biology and ecology to develop informed conservation and fisheries management policy.

The broadnose sevengill shark, *Notorynchus cepedianus* Peron, 1807, (Chondrichthyes: Hexanchidae) is a relatively common, large coastal species in many temperate marine ecosystems. It frequently inhabits bays and estuaries throughout the eastern North Pacific and many aspects of it’s biology and ecology has been studied [8, 9, 10]. Recently it was shown, using acoustic tagging methods, that these sharks migrate between summer and winter grounds returning seasonally to the same bays and estuaries thought to be driven as part of their reproductive cycle [9]. Even though much is known about their basic biology and life history characteristics, virtually nothing is known about their population genetics such as genetic diversity, genetic structure, and kin associations. To help fill in this data gap we investigated the genetic diversity and relatedness of *N*. *cepedianus* within Willapa Bay, Washington and San Francisco Bay, California. We used microsatellite markers developed within a related species within the family Hexanchidae, the bluntnose sixgill shark, *Hexanchus griseus* [11]. Here we ask three specific questions: 1) How much genetic diversity is found within *N*. *cepedianus*, 2) What is the population structure between these two sampled regions; and 3) What are the familial relationships among individuals within and between these populations.

## Materials and Methods

Samples for genetic analysis were small (approximately 2–3 mm) fin clips taken during hook and line or longline fishing operations. Samples were collected during 2005 by the Washington Department of Fish and Wildlife in Willapa Bay, Washington (WA, n = 44), from 2003–2007 by the Monterey Bay Aquarium (SF-1, n = 36), and from 2007–2012 by the Aquarium of the Bay within San Francisco Bay, California (SF-2, n = 53) for a total of 133 tissue samples. The size range for sharks sampled in San Francisco were a mixture of adults and subadults: males averaged 148 cm with 66% mature (mature size range for males is between 150 and 180 cm) and females averaged 133 cm with only 15% mature (mature size range for females is between 192 and 208 cm). The sharks sampled in Washington were also a mixture of adults and subadults and in general were larger than those sampled in San Francisco: males averaged 206 cm with 93% mature and females averaged 195.25 cm with 38% mature. Tissue samples from sharks were preserved in 70–100% ethanol and/or frozen at -20°C or -55°C until analysis. DNA was extracted from tissue using the DNeasy Blood and Tissue Kit (Qiagen, Valencia, California). Conditions for polymerase chain reaction (PCR) were optimized for amplification of the seven microsatellite loci (SG13, SG24, SG25, SG27, SG28, SG30 and SG31) [11]. Each microsatellite locus was amplified separately using a GeneAmp PCR 9600 thermal-cycler (Perkin Elmer, Wellesley, Massachusetts) in a total volume of 10μl containing 1μl of 100–250 ng/μl purified DNA template, 0.5 μM/μl forward and reverse primer, 4μl PCR Mastermix 2X (Taq polymerase with manufacturer’s supplied buffer, dNTPs and MgCl_2,_ Promega, Madison, Wisconsin), and 4μl DNA/RNA free dH_2_O. The amplification profile of each primer follows that described by Larson et al., 2011 [11]. PCR products were stored at 4°C or -20°C until analysis using an Applied BioSystems (ABI, Foster City, California) 3100 sixteen-capillary system in Genescan mode. Each run contained at least one reference sample as well as several repeated samples to ensure accuracy. There was no multiplexing of loci.

Allele scoring was performed using Genescan Software version 3.0 (Applied Biosystems) or Peakscanner Software 1.0 (Applied Biosystems). Tests for departures from Hardy-Weinberg equilibrium were performed using the Hardy-Weinberg probability function with default Markov chain parameters in Option One of GENEPOP 3.1 software [12]. Sequential Bonferroni adjustments over all loci were used to determine significance levels for all simultaneous tests, resulting in a final significant p value of ≤ 0.007 [13]. MICROCHECKER software [14] was used to determine genotyping errors such as allelic dropout, stuttering and null alleles.

Genetic diversity estimates, observed and expected heterozygosity (*H*_O_ and *H*_E_), allele diversity, and population structure analyses such as Principal Coordinates Analysis (PCoA) and population assignment cluster analysis were measured using GenAlEx 6.5 [15].

Relatedness within populations was examined using three programs: MLRELATE [16], COLONY [17] and COANCESTRY [18]. MLRELATE calculates the maximum likelihood estimates of relatedness and relationships from microsatellites. The program COLONY uses a maximum likelihood method to assign siblings and parentage using individual genotypes at all markers. It estimates a full- and half-sibling relationship, assigns parentage, and evaluates reproductive skew, or the estimated percentage of each potential parent’s contribution to offspring genotypes. For this analysis both male and female sevengill sharks were assumed to use a polygamous mating system. The program COANCESTRY employs seven relatedness estimators. We choose to use the triadic likelihood estimator (denoted as TrioML), as this likelihood method uses the genotypes of a triad of individuals in estimating pairwise relatedness reducing the chance of genes identical in state (IIS) being mistakenly inferred as identical by descent (IBD). The method allows for inbreeding and accounts for genotype errors in data such as null alleles.

Finally BOTTLENECK software [19] was used to determine if the populations had experienced a recent significant population bottleneck that may now be affecting diversity. Specifically the program tests for heterozygosity excess under Hardy-Weinberg equilibrium since allelic diversity is expected to decrease faster than observed heterozygosity. We chose to use the two-phase model (TPM) with 70% and 90% single-step mutations combined with a variance of 30 and the one-tailed Wilcoxon sign rank test to determine statistical significance.

## Results

**Fig 1:**
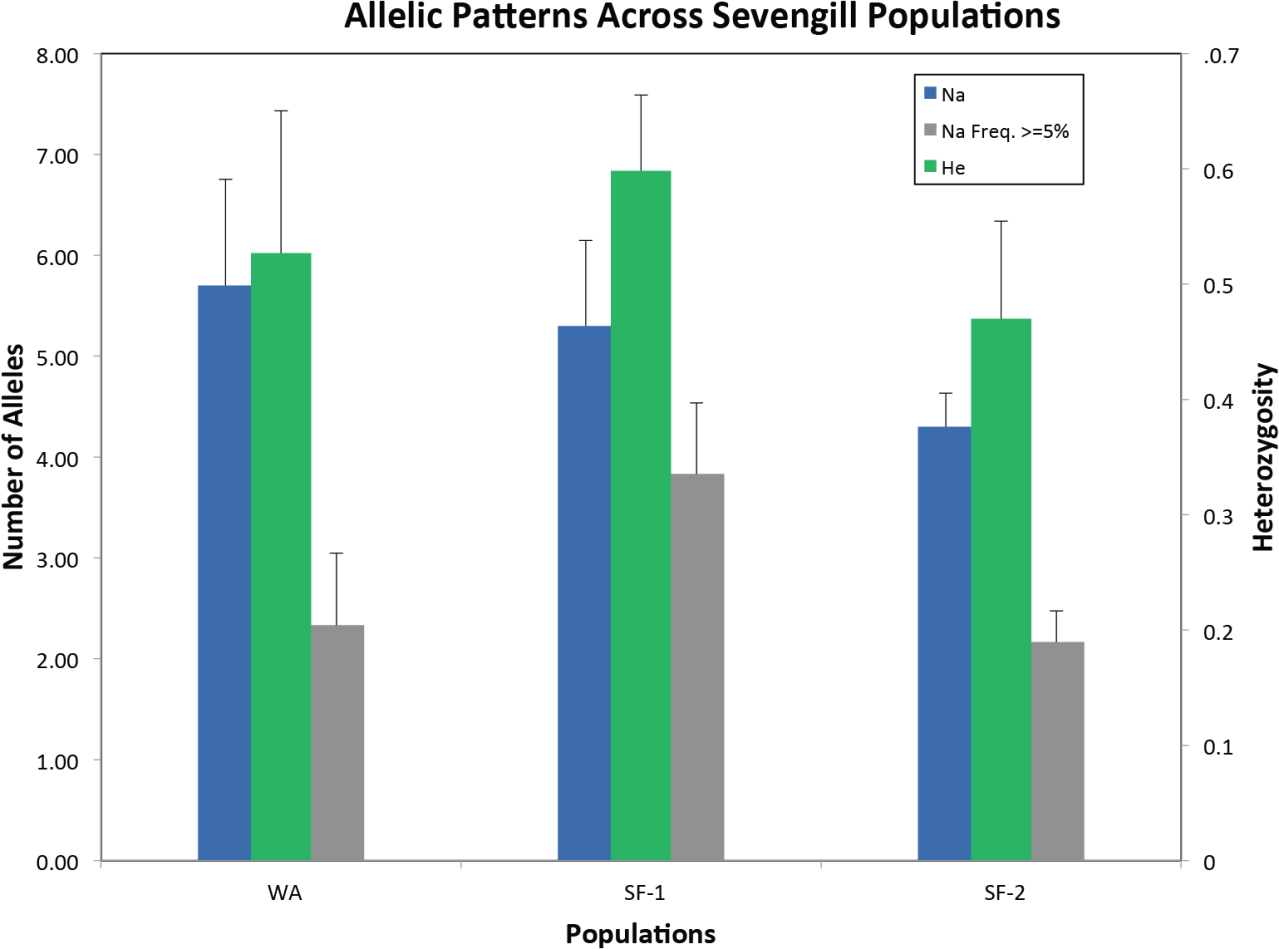
Allelic patterns and diversity among measured sevengill populations. NA = Average number of alleles and He = expected heterozygosity.

**Fig 2:**
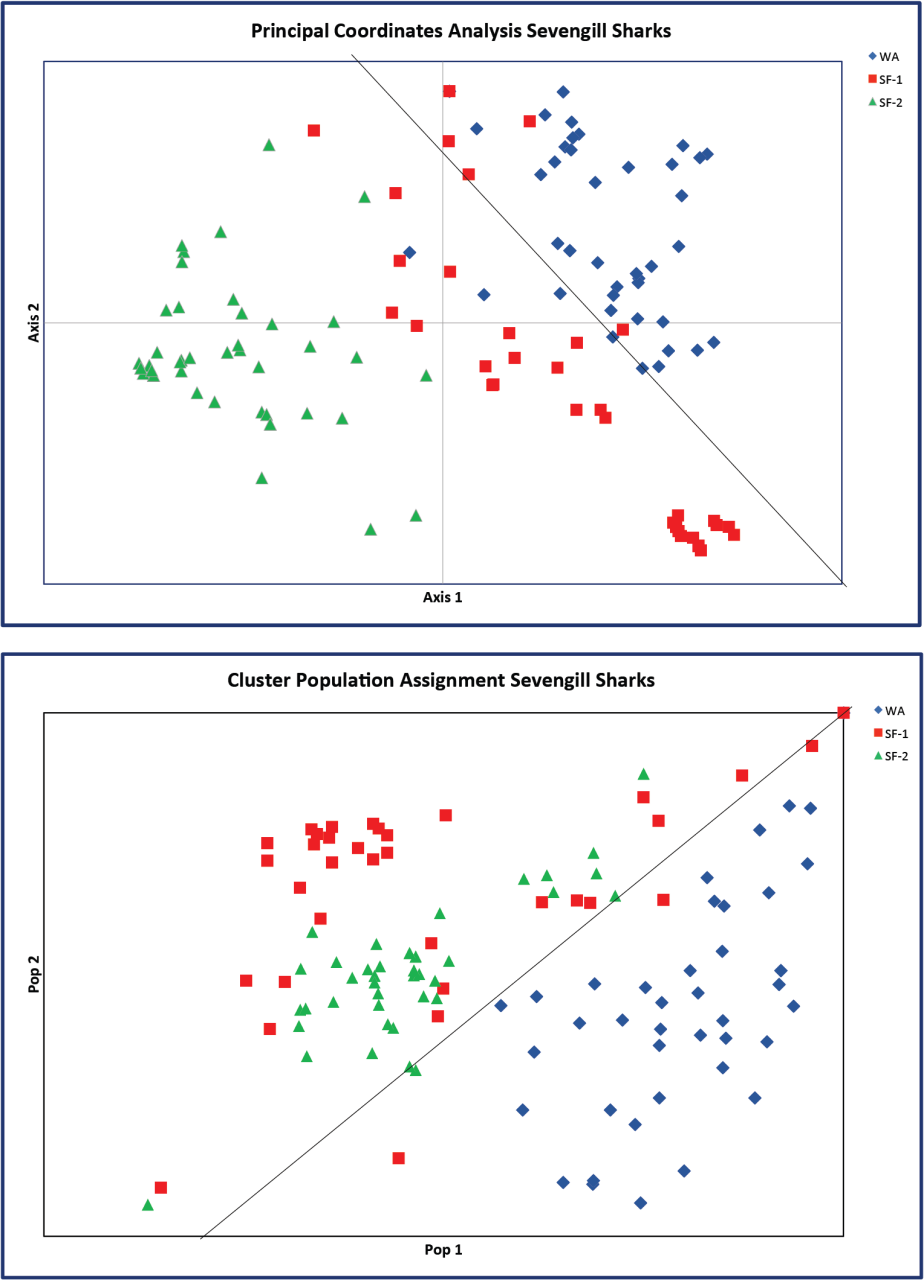
A: Principal Coordinates Analysis of Sevengill sharks. Axis 1 and 2 explaining 56% of the variation. Note WA and SF-2 clearly separated but WA and SF-1 intermixing which is likely due to some shared common ancestry. B: Population assignment of sevengill shark using cluster analysis. Note WA separating from the two SF groups.

Departures from Hardy Weinberg (H-W) expectations were not significant (Fis p values range = 0.0115–0.3412, Bonferroni corrected α = 0.007). There was no evidence of linkage disequilibrium and no evidence of genotyping errors as evaluated by GENEPOP 3.1 software [11]. MICROCHECKER software [14] indicated that there were null alleles within SG24, SG27 and SG31. Locus specific statistics as well as population diversity estimates are listed in Table 1. The average number of microsatellite alleles per locus was 5.1 (range: 4.0–7.0), the average observed heterozygosity (*H*_O_) was 0.417 (range, 0.171–0.593), and average expected heterozygosity (*H*_E_) was 0.531 (range, 0.444–0.634, Table 1). Average numbers of alleles, percent allele frequencies greater than 5%, and expected heterozygosity (*H*_E_) per population are shown graphically in Fig 1. Population structure was determined using multiple analyses within the software GeneAlEx (PCoA and population assignment cluster analysis, Figs 1 and 2). The Principal Coordinate Analysis (PCoA) suggests some similarity between the geographic areas with WA and SF-1 individuals mixing on one side of the graph and SF-2 on the other (Fig 2A). However the population assignment cluster analysis clearly separates the two geographic areas on either sides of the graph (Fig 2B). Assignment analysis found that only 1 individual within the WA population miss-assigned to the SF-1 population while 5 individuals within the SF-1 population miss-assigned (3 to WA and 2 to SF-2).

**Table 1:** Microsatellites amplified within sevengill sharks.

Locus	A	*H*_*E*_	*H*_*O*_	Fis	p	% null	GFR
SG13	7.0	0.634	0.593	-0.3636	0.0280	0.00	0.00
SG24	7.0	0.448	0.265	0.3786	0.0326	0.13	0.00
SG25	4.0	0.444	0.397	0.0276	0.1724	0.00	0.00
SG27	4.0	0.457	0.567	0.1711	0.3412	0.28	0.00
SG28	4.3	0.622	0.413	0.2228	0.1052	0.00	0.00
SG30	4.3	0.572	0.516	0.0163	0.2084	0.00	0.00
SG31	5.0	0.536	0.171	0.9540	0.0115	0.37	0.00
**AVE**	5.1	0.531	0.417			0.11	0.00

A = Number of alleles, *H*_*E*_ = expected heterozygosity, *H*_*O*_ = observed heterozygosity as described by GENALEX; Fis values, P value of Fis, and GFR = genotyping failure rate as described by GENEPOP; %null = % null alleles as described by MICROCHECKER.

Relatedness analysis using all three relatedness software programs showed a high degree of relatedness between sharks caught in the same geographic area and sampled in the same time period (Table 2). Average proportional first order relationships (e.g. full siblings: FS and half siblings: HS) of sharks using all statistical programs within geographic areas was 0.423 while average proportional first order relationships was 0.098 between geographic areas (Table 2). Proportional relatedness within populations using MLRELATE ranged from 0.329 within SF-1 to 0.732 within SF-2; using COANCESTRY relatedness ranged from 0.313 within WA to 0.717 within SF-2; and using COLONY relatedness ranged from 0.184 within SF-1 to 0.385 within SF-2 (Table 2). COLONY results suggested 24 different cohort groups among all *N*. *cepedianus* sampled, with nine cohorts in the Washington population and 15 cohorts found in California (7 in SF-1 and 8 in SF-2). Only one out of the 133 *N*. *cepedianus* miss-assigned to a population different than their origin; one individual from San Francisco Bay was assigned to a Washington cohort. Proportional relatedness between populations was significantly lower (p<0.0001) than that found within populations. Proportional relatedness between populations using MLRELATE ranged from 0.119 between SF-1 to 0.144 between WA and SF-2; using COANCESTRY ranged from 0.071 between WA and SF-1 to 0.162 between WA and SF-2; and using COLONY ranged from 0.028 between WA and SF-1 to 0.066 between WA and SF-2 (Table 2). These relationships are genetic probabilities and may or may not represent actual related individuals.

**Table 2:** Percentage relatedness (1 = 100%) within and between sevengill shark populations.

**MLRELATE**				
*Within*	FS	HS	U	%related
WA	0.354	0.130	0.509	0.484
SF-1	0.274	0.055	0.634	0.329
SF-2	0.537	0.195	0.268	0.732
*Between*				
SF-1+SF-2	0.097	0.022	0.881	0.119
WA+SF-1	0.117	0.023	0.860	0.140
WA+SF-2	0.131	0.013	0.856	0.144
**COANCESTRY**				
*Within*	FS	HS	U	%related
WA	0.212	0.101	0.688	0.313
SF-1	0.309	0.115	0.575	0.424
SF-2	0.512	0.205	0.282	0.717
*Between*				
SF-1+SF-2	0.072	0.048	0.881	0.120
WA+SF-1	0.036	0.035	0.93	0.071
WA+SF-2	0.115	0.047	0.838	0.162
**COLONY**				
*Within*	FS	HS	U	%related
WA	0.084	0.152	0.765	0.236
SF-1	0.094	0.090	0.816	0.184
SF-2	0.067	0.317	0.616	0.385
*Between*				
SF-1+SF-2	0.002	0.028	0.971	0.030
WA+SF-1	0.012	0.016	0.972	0.028
WA+SF-2	0.007	0.059	0.934	0.066
**Ave. within**	**0.271**	**0.151**	**0.573**	**0.423**
**Ave. between**	**0.065**	**0.032**	**0.903**	**0.098**

FS: Full sibling, HS: Half sibling, and U: Unrelated. The values from COLONY were significantly different from COANCESTRY and MLRELATE at the p = 0.05 level for related individuals within and between populations. The values of within vs between relatedness found in all three programs were significant at the p = 0.0001 level (paired t tests).

BOTTLENECK results suggest no recent population bottleneck. P values for the one-tailed Wilcoxon sign rank test for all populations were not significant (70% probability p value ranged from 0.594 to 0.988 and 90% probability p value ranged from 0.656 to 0.996).

## Discussion

The primary goal of this study was to explore the genetic diversity, population structure and relatedness within *N*. *cepedianus* in two estuaries in the northeast Pacific. We acknowledge the number of loci used was low and thus that the power of the analyses will also be low. However we feel that these preliminary results are compelling and hope that this work stimulates more genetic analyses of *N*. *cepedianus*.

The average genetic diversity *H*_*O*_ = 0.417 and *H*_*E*_ = 0.531 found within *N*. *cepedianus* was on the low end of the microsatellite diversity compared to other shark species (Table 3) [20–28]. All of the microsatellites used in this study were originally developed for another cowshark, *Hexanchus griseus*, and, as such, the low microsatellite diversity in *Notorynchus cepedianus* could be caused, in part, by ‘‘ascertainment bias,” the suggested tendency for microsatellites to be less variable in species other than those from which they were originally selected [29]. However, we found that the levels of heterozygosity reported here within *Notorynchus cepedianus* were still low when compared to those reported within shark species that employed loci developed from another species. For example Ovenden et al. (2009) [30] reported an average *H*_*E*_ of 0.76 in loci applied to four non-target species; Ovenden, et al. (2006) [31] reported *H*_*E*_ of 0.64 within one non-target species; and Chapman et al. (2004) [32] reported *H*_*E*_ of 0.68 within one non-target species (Table 3).

**Table 3:** Genetic diversity of microsatellite loci in sixteen elasmobranch species.

Species	*H*_*E*_ range	Source
***Carcharhinus limbatus***	0.68–0.95	Keeney et al. 2005
***Carcharhinus obscurus***	0.52–0.90	Ovenden et al. 2009*
***Carcharhinus plumbeus***	0.84–0.97	Portnoy et al. 2007
***Carcharhinus sorrah***	0.03–0.94	Ovenden et al. 2009*
***Carcharhinus sorrah***	0.16–0.82	Ovenden et al. 2006*
***Carcharhinus tilstoni***	0.54–0.92	Ovenden et al. 2006*
***Carcharias taurus***	0.47–0.81	Ahonen, Harcourt and Stow 2009
***Carcharodon carcharias***	0.45–0.95	Pardini et al. 2000
***Ginglymostoma cirratum***	0.17–0.90	Heist et al. 2002
***Hexanchus griseus***	0.08–0.90	Larson et al. 2011
***Negaprion brevirostris***	0.69–0.89	Feldheim, Gruber, and Ashley 2001
***Notorynchus cepedianus***	0.43–0.53	This study*
***Prionace glauca***	0.31–0.88	Ovenden et al. 2009*
***Rhincodon typus***	0.40–1.00	Schmidt et al. 2009
***Sphyrna lewini***	0.17–0.87	Ovenden et al. 2009*
***Sphyrna tiburo***	0.67	Chapman et al. 2004*
***Squalus mitsukurii***	0.35–0.86	Daly-Engel et al. 2010

* = indicates He range is for markers developed within other species.

The relatively low genetic variation reported here within *N*. *cepedianus* compared to other shark species, suggests potential past population bottlenecks that may now be affecting diversity. There was a targeted fishery for *N*. *cepedianus* in San Francisco Bay from the 1930’s to the 1980’s [33]. We employed BOTTLENECK software to determine if there was support for a historical population bottleneck potentially caused by the fishery. The non-significant results of the TMP model suggest that *N*. *cepedianus* did not experience a significant loss of diversity due to take from targeted fishing between 1930–1980. However there is evidence that the effectiveness of detecting bottlenecks using this software depends on the number of generations post bottleneck [34]. For example analyses conducted one to five generations post bottleneck event were found to have very low statistical power (<0.5) and analyses conducted after even 10 generations had only modest statistical power (0.6) [34]. The generation time of *N*. *cepedianus* is unknown, but is thought to be relatively long (between 10–15 years) [8]. Thus it is likely that only two to three generations of this species have occurred between 1980 and 2005 when sampling began and the power to detect the bottleneck this soon may be low. In addition the power of BOTTLENECK depends highly on the number of loci used [35]. The seven loci employed here is likely not a large enough sample size to accurately detect a bottleneck. We feel more work should be done to investigate the potential of a historical bottleneck affecting *N*. *cepedianus* diversity using more molecular markers.

Population comparisons between California and Washington *N*. *cepedianus* revealed a moderate to high degree of genetic structure (Principal Coordinates Analysis and population assignment, Fig 2). This structure could be due to *N*. *cepedianus* having relatively small home ranges with high site fidelity [35]. Although they display high seasonal site fidelity, they are also migratory, moving between specific regions on annual cycles for feeding, breeding, and/or pupping [9]. For example some of the Washington *N*. *cepedianus* were found to have well defined migration routes between summer/fall grounds (May-October) in Willapa Bay and Humboldt Bay in California where they were found the rest of the year [9]. In addition, many of the Willapa bay tagged animals were found in the same areas of Willapa Bay year after year, suggesting very high long-term site fidelity [9].

Based on the genetic structuring found between the California and Washington populations of *N*. *cepedianus* it is thought that these two groups may have separate breeding grounds. This is the case in other species of sharks that exhibit relatively high stock structure, such as *Carcharhinus limbatus* in the Gulf of Mexico and Atlantic [36]. In this study female *C*. *limbatus* did not disperse randomly between regions during migrations to nursery areas and related females tended to return to the same region. A similar pattern may hold true for female *N*. *cepedianus* sampled here: perhaps these sharks travel in sexually segregated and related groups to separate nursery and breeding grounds. As within *C*. *limbatus* there is most likely migration of females and males between regions resulting in limited gene flow, as suggested by some of the SF-1 *N*. *cepedianus* intermixing with the WA population in the PCoA and the assignment plot (Fig 2). The mixing shown between WA and SF-1 could be in part because these two groups were sampled during the same years (2005) and some individuals may be the progeny of the same effective breeding group.

One of the most interesting findings of this preliminary study was the high degree of relatedness among *N*. *cepedianus* caught in each area and during similar time periods (Table 2). The relatedness program results varied in the amount of related individuals suggested within groups; with MLRELATE and COANCESRY producing similar results and COLONY suggesting the lowest levels of relatedness. All three programs resulted in significantly higher within population relatedness than between population relatedness suggesting a real pattern rather than an artifact of the low number of loci and diversity. In addition the sampling of several sharks at the same time and place, which was known to occur in both Washington and California, may have biased the results by sampling a high number of related individuals. High levels of relatedness in sharks sampled at the same time and location has been documented in other elasmobranchs such as *Negaprion brevirostris* and *Hexanchus griseus* [11, 37] One theory for this behavior is that newborn sharks recruit to a natal area in cohort groups and remain relatively tightly associated until adulthood [11]. Such associations may benefit individuals such as finding food and engaging in cooperative foraging, a behavioral trait noted to occur in *N*. *cepedianus* [38].

Several recent studies have documented kin association in the aquatic environment [11, 39–44]. Over half the studies found high levels of relatedness in fish associated in time and space, such as black perch *Embiotoca jacksoni* [44], humbug damselfish *Dascyllus aruanus* [43], kelp bass *Paralabrax clathratus* [41], and Atlantic salmon *Salmo salar* [40]. This might be expected for the species that are viviparous such as *E*. *jacksoni*, or have known migration routes such as *S*. *salar*, and may facilitate effective dispersal of the genes shared within the related group by increasing the odds that any single individual survives [40, 44]. There may be other adaptive benefits for these fish staying in related groups, such as increased foraging success or an increased ability to detect predators [45–47].

*Notorynchus cepedianus* occupies shallow, near shore estuaries such as San Francisco Bay in California and Willapa Bay in Washington during at least part of the year and has historically been targeted by fisheries [9, 10]. This preliminary study observed a high number of related individuals sampled within the same time and space adding to the growing body of knowledge regarding associations of related individuals in the marine environment. The presence of estuary-specific familial relationships suggests that conservation measures and fishery management policies for *N*. *cepedianus* on the U.S. West Coast should incorporate geographically explicit, population-specific goals to ensure the long-term persistence of this ecologically important predator.

## References

[1] Hoenig JM, Gruber SH (1990) Life-history patterns in the elasmobranchs: implications for fisheries management. In: Pratt HL Jr., Gruber SH, Taniuchi T, editors. Elasmobranchs as living resources: advances in biology, ecology, systematics and status of the fisheries. NOAA Technical Report NMFS 90. pp. 1–16.

[2] Musick JA, Burgess G, Cailliet G, Camhi M, Fordham S (2000) Management of sharks and their relatives (Elasmobranchii). Fisheries 25: 9–13.

[3] Schindler DE, Essington TE, Kitchell JF, Boggs C, Hilborn R (2002) Sharks and tunas: fisheries impacts on predators with contrasting life histories. Ecol Appl 12 (3):735–748.

[4] Baum JK, Myers RA, Kehler DG, Worm B, Harley SJ, Doherty PA (2003) Collapse and conservation of shark populations in the Northwest Atlantic. Science 299:389–392. 12532016 10.1126/science.1079777

[5] Baum JK, Myers RA (2004) Shifting baselines and the decline of pelagic sharks in the Gulf of Mexico. Ecol Lett 7:135–145.

[6] Dulvy NK, Fowler SL, Musick JA, Cavanagh RD, Kyne PM, Harrison LR, et al. (2014) Extinction risk and conservation of the world’s sharks and rays. eLife 10.7554/eLife.00590 PMC3897121 24448405

[7] IUCN. (2014) http://www.iucn.redlist.org. Accessed Oct 2014.

[8] Ebert DA (1989) Life history of the sevengill shark, *Notorynchus cepedianus*, Peron 1807, in two northern California bays. Calif Fish Game 75(2): 102–112.

[9] Williams GD, Andrews KS, Katz SL, Moser ML, Tolimieri N, Farrer DA, et al (2012) Scale and pattern of broadnose sevengill shark *Notorynchus cepedianus* movement in estuarine embayments. J Fish Biol 80(5):1380–1400. 10.1111/j.1095-8649.2011.03179.x 22497389

[10] Williams GD, Andrews KS, Farrer DA, Bargmann GG, Levin PS (2011) Occurrence and biological characteristics of broadnose sevengill sharks (*Notorynchus cepedianus*) in Pacific Northwest coastal estuaries. Environ Biol Fish 91: 379–388.

[11] Larson S, Christiansen J, Griffing D, Ashe J, Lowry D, Andrews K (2011) Relatedness and polyandry of sixgill sharks, *Hexanchus griseus*, in an urban estuary. Conserv Genet 12: 679–690. 10.1007/s10592-010-0174-9

[12] Raymond M, Roussett F (1995) Genepop (Version 1.2): Population genetics software for exact tests and ecumenicism. J Hered 86:248–249.

[13] Rice WR (1989) Analyzing tables of statistical tests. Evolution 43:223–225. 28568501 10.1111/j.1558-5646.1989.tb04220.x

[14] Oosterhout CW, Hutchinson B, Wills D, Shipley P (2004) MICRO-CHECKER: software for identifying and correcting genotyping errors in microsatellite data. Mol Ecol Notes 4: 535–538

[15] Peakall R, Smouse PE (2006) GenAlEx 6: genetic analysis in Excel. Population genetic software for teaching and research. Mol Ecol Notes 6: 288–295. 10.1093/bioinformatics/bts460 PMC3463245 22820204

[16] Kalinowski ST, Wagner AP, Taper ML (2006) ML-Relate: a computer program for maximum likelihood estimation of relatedness and relationship. Mol Ecol Notes 6:576–579.

[17] Wang J (2004) Sibship reconstruction from genetic data with typing errors. Genetics 166: 1963–1979. 15126412 10.1534/genetics.166.4.1963 PMC1470831

[18] Wang J (2011) COANCESTRY: A program for simulating, estimating and analysing relatedness and inbreeding coefficients. Molecular Ecology Resources 11: 141–145. 10.1111/j.1755-0998.2010.02885.x 21429111

[19] Piry S, Luikart G, Cornuet JM (1999) BOTTLENECK: A computer program for detecting recent reductions in the effective population size suing allele frequency data. 90 (4): 502–503.: 10.1093/jhered/90.4.502

[20] Keeney DB, Heupel MR, Hueter RE, Heist EJ (2005) Microsatellite and mitochondrial DNA analyses of the genetic structure of blacktip shark (*Carcharhinus limbatus*) nurseries in the northwestern Atlantic, Gulf of Mexico, and Caribbean Sea. Mol Ecol 14:1911–1923. 15910315 10.1111/j.1365-294X.2005.02549.x

[21] Heist EJ, Gold JR (1999) Micosatellite DNA variation in sandbar sharks (*Carcharhinus plumbeus*) from the Gulf of Mexico and Mid-Atlantic Bight. Copeia 1:182–186.

[22] Portnoy DS, Piercy AN, Musick JA, Burgess GH, Graves JE (2007) Genetic polyandry and sexual conflict in the sandbar shark, *Carcharhinus plumbeus*, in the western North Atlantic and Gulf of Mexico. Mol Ecol 16:187–197. 17181730 10.1111/j.1365-294X.2006.03138.x

[23] Feldheim KA, Gruber SH, Ashley MV (2001) Population genetics structure of the lemon shark (*Negaprion brevirostris*) in the western Atlantic: DNA microsatellite variation. Mol Ecol 10:295–303. 11298946 10.1046/j.1365-294x.2001.01182.x

[24] Heist EJ, Jenkot JL, Keeney DB, Lane RL, Moyer GR, Reading BJ, et al (2002) Isolation and characterization of polymorphic microsatellite loci in nurse shark (*Ginglymostoma cirratum*). Mol Ecol Notes 3:59–61.

[25] Pardini AT, Jones CS, Scholl MC, Noble LR (2000) Isolation and characterization of dinucleotide microsatellite loci in the Great White Shark, *Carcharodon carcharias* . Mol Ecol 9(8):1176–1178. 10964240 10.1046/j.1365-294x.2000.00954-4.x

[26] Ahonen H., Harcourt RG, Stow AJ (2009) Nuclear and mitochondrial DNA reveals isolation of imperiled grey nurse shark populations (*Carcharias taurus*) Mol Ecol 18: 4409–4421. 10.1111/j.1365-294X.2009.04377.x 19804378

[27] Schmidt JV, Schmidt CL, Ozer F, Ernst RE, Feldheim KA, Ashley MV, et al (2009) Low Genetic Differentiation across Three Major Ocean Populations of the Whale Shark, *Rhincodon typus* . PLoS ONE (4): e4988 10.1371/journal.pone.0004988 19352489 PMC2662413

[28] Daly-Engel TS, Grubbs RD, Feldheim KA, Bowen BW, Toonen RJ (2010) Is multiple mating beneficial or unavoidable? Low multiple paternity and genetic diversity in the shortspine spurdog *Squalus mitsukurii* . Mar Eco Prog Ser 403:255–267.

[29] Ellegren H, Primmer CR, Sheldon B (1995). Microsatellite evolution: directionality or bias in locus selection? Nat Genet 11: 60–62. 7493011 10.1038/ng1295-360

[30] Ovenden JR, Kashiwagi T, Broderick D, Giles J, Salini J (2009) The extent of population genetic subdivision differs among four co-distributed shark species in the Indo-Australian archipelago BMC Evol Biol 9: 40 10.1186/1471-2148-9-40 19216767 PMC2660307

[31] Ovenden JR, Broderick D, Street R (2006) Microsatellite primers for two carcharinid sharks (*Carcharinus tilstoni* and *C*. *sorrah*) and their usefulness across a wide range of shark species. Mol Ecol Notes 6:415–418.

[32] Chapman DD, Prodohl PA, Gelsleichter J, Manire CA, Shivji MS (2004) Predominance of genetic monogamy by females in a hammerhead shark, *Sphyrna tiburo*: implications for shark conservation. Mol Ecol 13:1965–1974. 15189217 10.1111/j.1365-294X.2004.02178.x

[33] Ebert DA (2001) Cowsharks In: Leet WS, Dewees CM, Klingbiel R, Larson EJ, editors. California’s Living Marine Resources: A Status Report. The Resources Agency, California Department Fish and Game pp. 470–471.

[34] Peery MZ, Kirby R, Reid BN, Stoelting R, Doucet-Bëer E, Robinson S, et al (2012) Reliability of genetic bottleneck tests for detecting recent population declines. Mol Ecol 2114: 3403–3418 10.1111/j.1365-294X.2012.05635.x 22646281

[35] Ebert DA (2003) Sharks, rays and chimeras of California University of California Press Berkeley, California. 284 p.

[36] Keeney DB, Heupel MR, Hueter RE, Heist EJ (2003) Genetic heterogeneity among blacktip shark, *Carcharhinus limbatus*, continental nurseries along the U.S. Atlantic and Gulf of Mexico. Mar Biol 143: 1039–1046.

[37] Feldheim KA, Gruber SH, Ashley MV (2004) Reconstruction of parental microsatellite genotypes reveals female polyandry and philopatry in the lemon shark, *negaprion brevirostris* . Evolution 58(10): 2332–2342. 15562694 10.1111/j.0014-3820.2004.tb01607.x

[38] Ebert DA (1991) Observations on the predatory behaviour of the sevengill shark, *Notorynchus cepedianus* . S Afr J Marine Sci 11: 455–465.

[39] Planes S, Bonhomme F, Galzin R (1993) Genetic structure of *Dascyllus aruanus* populations in French Polynesia. Mar Biol 117:665–674.

[40] Olsén KH, Petersson E, Ragnarsson B, Lundqvist H, Jarvi T (2004) Downstream migration in Atlantic salmon (*Salmo salar*) smolt sibling groups. Can J Fish Aquat Sci 61(3): 328–331.

[41] Selkoe KA, Gaines SD, Caselle JE, Warner RR. (2006) Current shifts and kin aggregation explain genetic patchiness in fish recruits. Ecology 87:3082–3094. 17249233 10.1890/0012-9658(2006)87[3082:csakae]2.0.co;2

[42] Miller-Sims VC, Gerlach G, Kingsford MJ, Atema J (2008) Dispersal in the spiny damselfish, *Acanthochromis polyacanthus*, a coral reef fish species without a larval pelagic stage. Mol Ecol 17:5036–5048. 10.1111/j.1365-294X.2008.03986.x 19120989

[43] Buston PM, Fauvelot C, Wong MYL, Planes S (2009) Genetic relatedness in groups of the humbug damselfish, *Dascyllus aruanus*: small, similar-sized individuals may be close kin. Mol Ecol 18(22): 4707–4715. 10.1111/j.1365-294X.2009.04383.x 19845858

[44] Sikkel PC, Fuller CA (2010) Shoaling preference and evidence for maintenance of sibling groups by juvenile black perch (*Embiotoca jacksoni*). J Fish Biol 76:1671–1681. 10.1111/j.1095-8649.2010.02607.x 20557623

[45] Hamilton WD (1963) The evolution of altruistic behavior. Am Nat 97:354–356.

[46] Hamilton WD (1964) The genetical evolution of social behaviour, I and II. J Theor Biol 7:1–52. 5875341 10.1016/0022-5193(64)90038-4

[47] Griffin AS, West SA (2003) Kin discrimination and the benefit of helping in cooperatively breeding vertebrates. Science 302: 634–636. 14576431 10.1126/science.1089402

